# Comparison of efficacy and safety between long-acting growth hormone and short-acting growth hormone in isolated growth hormone deficiency: a systematic review and meta-analysis of 16 randomized controlled trials involving 2,435 pediatric patients

**DOI:** 10.3389/fped.2026.1776186

**Published:** 2026-06-04

**Authors:** Zhen-Zhen Shen, Yun Huang, Li-Li Wu, Jian-Su Zhang, Xiao-Xia Zhang

**Affiliations:** 1 Department of Pediatrics, Shanghai Xuhui District Central Hospital, Shanghai, China; 2 Department of Pediatrics, Tongde Hospital of Zhejiang Province, Hangzhou, China

**Keywords:** growth hormone deficiency (GHD), long-acting human growth hormone, meta-analysis, randomized controlled trials, short-acting daily growth hormone

## Abstract

**Objective:**

To comprehensively compare the efficacy and safety of long-acting human growth hormone (LAGH) preparations with conventional short-acting daily growth hormone (SA-GH) in children with isolated growth hormone deficiency (GHD), and explore potential differences across four distinct LAGH platforms through subgroup analyses.

**Methods:**

A systematic review and meta-analysis of randomized controlled trials (RCTs) published up to September 2025 was conducted. Five electronic databases [PubMed, Cochrane Library, Web of Science, WanFang Data, and China National Knowledge Infrastructure (CNKI)] were systematically searched without language restrictions. Eligible studies included patients aged <18 years with confirmed GHD (peak GH <10 ng·mL^−1^) and compared any approved once-weekly LAGH preparation with daily SA-GH. Primary outcomes were changes from baseline in height velocity standard deviation score (HV-SDS) and height standard deviation score (Ht-SDS); secondary outcomes included insulin-like growth factor-1 (IGF-1) SDS and adverse events (AEs). Subgroup analyses were performed based on LAGH platform (PEG-LAGH, somatrogon, somapacitan, lonapegsomatropin) and treatment duration.

**Results:**

A total of 16 RCTs involving 2,435 children (median follow-up: 52 weeks) were included. Overall, compared with SA-GH, LAGH resulted in a modest but statistically significant improvement in first-year Ht-SDS (mean difference [MD]: 0.08; 95% credible interval [CrI]: 0.04–0.11). Numerically, LAGH showed superior HV-SDS compared with SA-GH (MD: 0.85; 95% CrI: −0.39 to 2.09), an effect entirely driven by the PEG-LAGH subgroup (MD: 4.35; 95% CrI: 3.78–4.92). IGF-1 SDS was consistently higher in the LAGH group (MD: 0.51; 95% CrI: 0.21–0.80). AE rates did not differ significantly between groups (LAGH: 31%–46% vs. SA-GH: 35%–50%); the PEG-LAGH subgroup exhibited the lowest AE incidence (31.1%).

**Conclusions:**

Among children with isolated GHD, LAGH represents the preferred platform, offering both superior first-year height velocity and acceptable safety. In contrast, newer prodrug or albumin-binding LAGH formulations provide injection convenience but lack superior growth efficacy.

## 1 Introduction

Growth hormone deficiency (GHD) is one of the most common endocrine causes of childhood short stature, resulting from insufficient growth hormone secretion by the anterior pituitary gland and leading to impaired linear growth (1, 2). Without timely intervention, affected children may attain a lifelong adult height below −2 standard deviation scores (SDS) and experience psychosocial dysfunction (3, 4). Since the introduction of recombinant human growth hormone (rhGH) in 1985, standardized daily subcutaneous injections of short-acting rhGH (SA-GH) have been proven to significantly improve growth velocity (GV) and final adult height (AH) in children with GHD.However, adherence barriers associated with daily injections—including pain, psychological resistance, and forgetfulness—prevent 10%–30% of patients receiving long-term treatment from reaching their genetic target height (5–7).

In recent years, the development of long-acting growth hormone (LAGH) has emerged as a promising solution to address this challenge.Innovative platforms, represented by polyethylene glycol (PEG) technology, enable weekly or even biweekly administration via controlled-release mechanisms (8, 9). The released growth hormone, with a natural amino acid sequence, exhibits biological activity and tissue distribution consistent with endogenous growth hormone, significantly reducing injection frequency and theoretically improving treatment adherence and quality of life in pediatric patients (10, 11). A study from the Chinese CGLS database involving 339 GHD cases treated with PEG-rhGH showed that after five years of treatment, the mean Ht-SDS increased from −2.4 at baseline to −0.3, with a ΔHt-SDS of 2.1 ± 0.9. The AE incidence rate was 46.6% (predominantly mild infections, accounting for 29.8%), and only 1.0% of events were severe adverse events (SAEs), all unrelated to the medication (12). No PEG-rhGH antibodies were detected, and long-term safety remained stable, with AE rates decreasing annually over time. In addition to PEG-rhGH, currently approved or late-stage clinical LAGH candidates worldwide include lonapegsomatropin, somatrogon, and somapacitan. However, significant differences in molecular structure, pharmacokinetic characteristics, dose conversion, and immunogenicity among different LAGH platforms result in inconsistent therapeutic efficacy and safety profiles (13–15). While previous meta-analyses have attempted to compare the efficacy and safety of LAGH and daily SA-rhGH, most studies have limitations: lack of subgroup analyses across different LAGH platforms, over-reliance on populations of European and American descent, and insufficient external validation with Asian pediatric data. Furthermore, with the publication of multiple new Phase III clinical trials and real-world study results, there is an urgent need to update evidence-based knowledge to guide clinical practice (16–19).

Therefore, this study aimed to systematically search and synthesize relevant RCTs published before September 2025 in accordance with the PRISMA 2020 statement. It compares the efficacy (including changes in HV-SDS, Ht-SDS, and IGF-1 SDS) and safety of different LAGH formulations with SA-rhGH in the treatment of children with GHD, while conducting subgroup analyses based on LAGH platform and treatment duration. This study seeks to provide clinicians with high-quality, personalized evidence-based guidance for selecting growth hormone formulations, and to offer data support for the future optimization and development of LAGH preparations.

## 2 Methods

### 2.1 Study design and protocol registration

This systematic review and pairwise meta-analysis was conducted in accordance with the Preferred Reporting Items for Systematic Reviews and Meta-Analyses (PRISMA) 2020 statement and was prospectively registered prospectively registered in PROSPERO (registration number: CRD420251274065). Since only anonymized, published data were analyzed, ethical committee approval was waived.

### 2.2 Search strategy

We systematically searched PubMed (MEDLINE), Cochrane Library, Web of Science, WanFang Data, and CNKI from their inception to September 2025, without language restrictions. Studies published in languages other than English were screened using translated titles and abstracts (via Google Translate for initial screening, followed by professional translation for full-text review when necessary). Specifically, Chinese-language studies from WanFang Data and CNKI were screened by two bilingual investigators (Z.S. and Y.H.) who are native Chinese speakers. No studies were excluded based on language alone.The search was guided by the PICOS framework using the terms: “long-acting growth hormone” AND “short-acting growth hormone” AND “growth-hormone deficiency” AND “children/adolescents”. Reference lists of all eligible articles and related reviews were hand-searched to identify additional studies.

### 2.3 Eligibility and exclusion criteria

Eligibility criteria: Randomized controlled trials (RCTs), quasi-RCTs; children aged <18 years with confirmed GHD (peak GH <10 ng·mL^−1^ on two stimulation tests), baseline height SDS ≤–2.0, and no additional pituitary hormone deficits; once-weekly administration of long-acting recombinant human GH (LA-rhGH) vs. conventional daily SA-rhGH.

Exclusion criteria: Crossover trials without first-period data; mixed etiology cohorts lacking GHD subgroup data; abstracts without sufficient numerical results; and non-original articles (editorials, reviews, case reports).

### 2.4 Study selection

**Figure 1:**
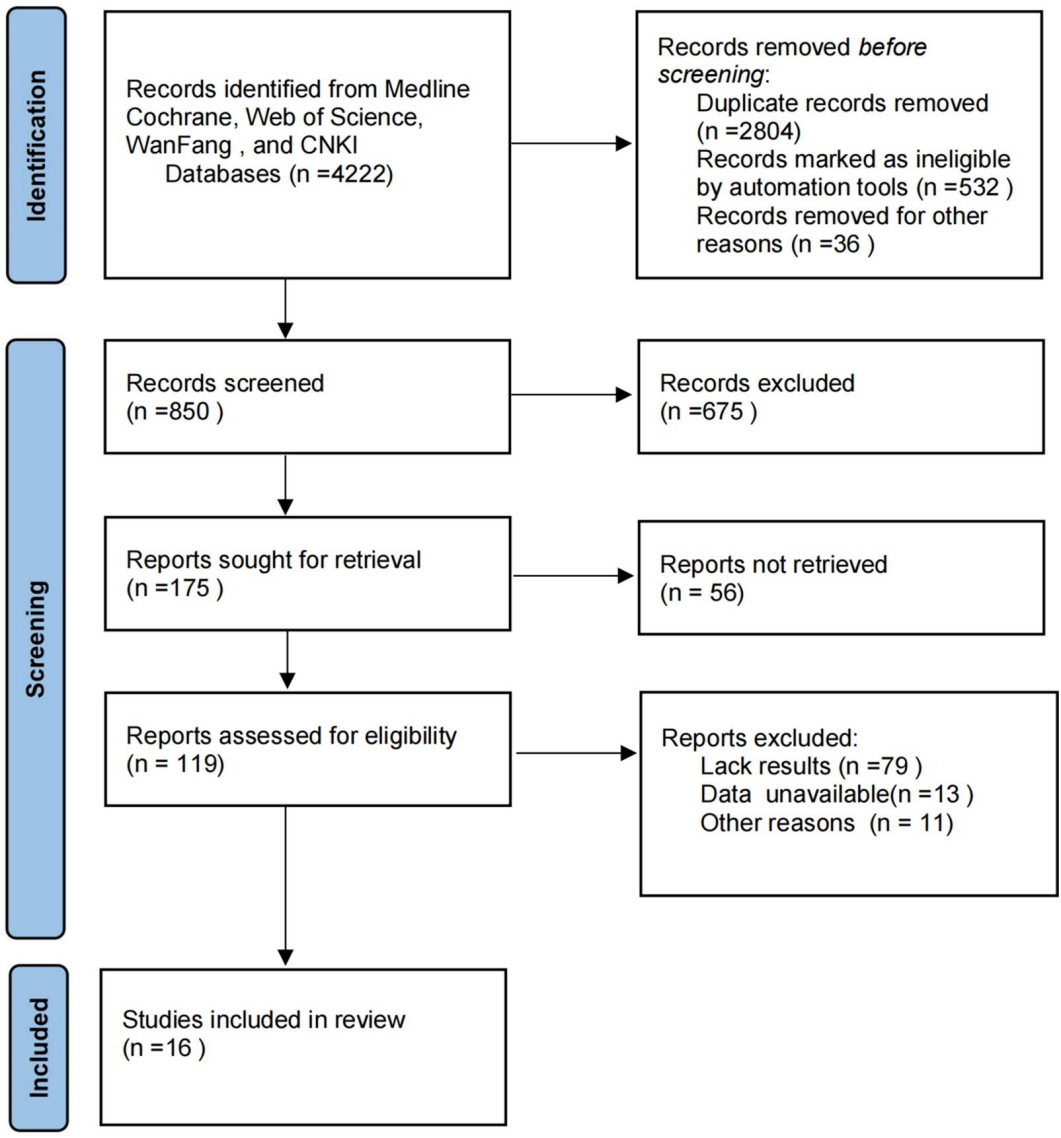
PRISMA flow diagram.

After duplicate removal using EndNote 21, titles and abstracts were independently screened in a masked manner by two investigators (Y.H. and J.Z.) using Rayyan. Full texts were evaluated against the eligibility checklist independently by the same two investigators. Data extraction was performed independently by two investigators (Y.H. and L.W.) using a pilot-tested, standardized Excel form.Disagreements were resolved by consensus; persistent discrepancies were adjudicated by a third author (Z.S.). A PRISMA flow diagram summarizes the screening process (Figure 1).

### 2.5 Data extraction

A pilot-tested, standardized Excel form was used to extract data independently by two investigators (Y.H. and L.W.), including: bibliographic details, study design, country of origin, participant characteristics (sample size, pubertal status, GH dose, treatment duration), outcome data at predefined time points, and methodological items for risk-of-bias assessment. Corresponding authors were contacted up to twice (with a 4-week interval) to request missing information. Continuous outcomes reported as medians with ranges were converted to means and standard deviations using the Luo-Wan method.

### 2.6 Risk-of-bias assessment

Risk of bias in included RCTs was assessed using the revised Cochrane Risk-of-Bias tool for randomized trials (RoB 2), which evaluates five domains: (1) randomization process, (2) deviations from intended interventions, (3) missing outcome data, (4) measurement of the outcome, and (5) selection of the reported result. Each domain was rated as low risk, some concerns, or high risk. An overall risk-of-bias judgment was assigned based on the most severe rating across domains. Two independent reviewers (Y.H. and L.W.) performed assessments, with disagreements resolved by consensus or adjudication by a third reviewer (Z.S.).

Additionally, Jadad scores (range: 1–5) were retained for sensitivity analyses only, to ensure comparability with prior meta-analyses. All primary risk-of-bias reporting and interpretation are based on RoB 2 assessments.

### 2.7 Data synthesis and statistical analysis

All analyses were performed using R software (v4.3.0). A Bayesian random-effects model was prespecified to pool effect estimates using the brms package with weakly informative priors. Continuous outcomes are presented as mean differences (MD) with 95% credible intervals (CrI); dichotomous outcomes as rate ratios with 95% CrI. *P* < 0.10 was considered indicative of small-study effects. If asymmetry was detected, the Duval & Tweedie trim-and-fill method was applied, and the adjusted estimate was reported.

#### 2.7.1 Assessment of heterogeneity

Heterogeneity was quantified using the posterior distribution of between-study variance (*τ*^2^) and Bayesian *I*^2^ statistics. We interpreted *I*^2^ values according to the Cochrane Handbook: 0%–40% as low heterogeneity, 30%–60% as moderate, 50%–90% as substantial, and 75%–100% as considerable heterogeneity. When substantial heterogeneity (*I*^2^ > 50%) was detected, we conducted subgroup analyses by LAGH platform and treatment duration, and meta-regression to explore potential sources of heterogeneity, including baseline Ht-SDS, mean age, GH dose, and geographic region. We also performed sensitivity analyses by excluding studies with high risk of bias (RoB 2 overall judgment) and by using different prior distributions.

#### 2.7.2 Assessment of publication bias

Publication bias was evaluated using comparison-adjusted funnel plots and Egger's regression test for each outcome, with *P* < 0.10 considered indicative of small-study effects. If funnel plot asymmetry was detected, we applied the Duval & Tweedie trim-and-fill method and reported adjusted estimates. We also examined contour-enhanced funnel plots to distinguish publication bias from other causes of asymmetry. Given that we included fewer than 10 studies for some outcomes, we interpreted funnel plot results cautiously, as test power is limited with small numbers of studies.

## 3 Results

A total of 16 eligible RCTs involving 2,435 children with GHD were included in the quantitative synthesis (Figure 1). Trials were conducted across Europe, North America, Japan, China, Egypt, Israel, Australia, and New Zealand between 2017 and 2024. Eight RCTs evaluated PEGylated long-acting rhGH (PEG-LAGH), four evaluated somatrogon, three evaluated somapacitan, and two evaluated lonapegsomatropin (TransCon). The mean age of participants ranged from 7.1 ± 2.3 to 11.4 ± 2.7 years; baseline Ht-SDS varied from −3.1 ± 0.7 to −2.0 ± 0.5. Follow-up duration spanned 26–208 weeks. The overall methodological quality was moderate (median Jadad score: 2; range: 2–4) (Table 1).

**Table 1:** Essential features of the randomized controlled studies in children with GHD.

Study ID	Countries	Design	Patients randomized (N)	Follow-up (weeks)	LAGH*	LAGH—Drug	Daily GH	daily GH—Dose	JADAD Score
Chatelain et al. (31)	Europe, Egypt	RCT phase 2	I: 40; C: 13	26	TransCon (ACP-001)	0.14, 0.21, 0.30 mg/kg/wk	Genotropin	0.34 mg/kg/wk	2
Deal et al. (32)	Worldwide	RCT phase 3	I: 109; C: 115	52	Somatrogon (MOD-4023)	0.66 mg/kg/wk	Genotropin	0.34 mg/kg/wk	4
Du et al. (33)	China	RCT phase 4	I: 48; C: 23	52	PEG-LAGH	0.12 mg/kg/wk; 0.20 mg/kg/wk	Genotropin	0.28 mg/kg/wk	2
Horikawa et al. (34)	Japan	RCT phase 3	I: 22; C: 22	52	Somatrogon (MOD-4023)	0.66 mg/kg/wk	Genotropin	0.025 mg/kg/wk	3
Luo et al. (35)	China	RCT phase 2/3	I: 228 (phase 2), 115 (phase 3); C: 115 (phase 3)	25	PEG-LAGH	0.1 mg/kg; 0.20 mg/kg/wk	Genotropin	0.25 mg/kg/wk	4
Maniatis et al. (36)	Europe, U.S.A., NZ	RCT phase 3	I: 103; C: 55	104	Lonapegsomatropin (Transcon)	0.24 mg/kg/wk	Genotropin	0.24 mg/kg/wk	4
Miller et al. (37)	USA, UK, Japan	RCT phase 3/4	I: 132; C: 68	52	Somapacitan	0.16 mg/kg/wk	Genotropin	0.24 mg/kg/wk	2
Miller et al. (38)	Europe, U.S.A., Asia	RCT phase 3	I: 132; C: 68	104	Somapacitan	0.16 mg/kg/wk	Soma	0.34 mg/kg/wk	3
Sävendahl et al. (24)	Europe, U.S.A., Japan	RCT phase 2	I: 43; C: 14	208	Somapacitan	0.04, 0.08, 0.16 mg/kg/wk	Norditropin	0.34 mg/kg/wk	2
Sävendahl et al. (39)	Europe, U.S.A., Japan	RCT phase 2	I: 43; C: 14	156	Somapacitan	0.04, 0.08, 0.16 mg/kg/wk	Norditropin	0.34 mg/kg/wk	2
Sun et al. (40)	China	RCT phase 4	I: 372; C: 176	26	PEG-LAGH	0.20 mg/kg/wk	Genotropin	0.25 mg/kg/wk	3
Thornton et al. (41)	Europe, U.S.A., NZ	RCT phase 3	I: 105; C: 56	52	Lonapegsomatropin (Transcon)	0.24 mg/kg/wk	Genotropin	0.34 mg/kg/wk	2
Chen and Jie (42)	China	RCT phase 4	I: 112; C: 126	24	PEG-LAGH	0.20 mg/kg/wk	Genotropin	0.25 mg/kg/wk	2
Zhang et al. (43)	China	RCT phase 4	I: 50; C: 60	24	PEG-LAGH	0.20 mg/kg/wk	Genotropin	0.25 mg/kg/wk	2
Wan et al. (44)	China	RCT phase 4	I: 20; C: 30	24	PEG-LAGH	0.20 mg/kg/wk	Genotropin	0.25 mg/kg/wk	2
Zelinska et al. (45)	Europe, U.S.A., Israel	RCT phase 2	I: 42; C: 11	52	Somatrogon (MOD-4023)	0.25, 0.48, 0.66 mg/kg/wk	Genotropin	0.34 mg/kg/wk	2

### 3.1 Height-velocity SDS (HV-SDS) change from baseline

**Figure 2:**
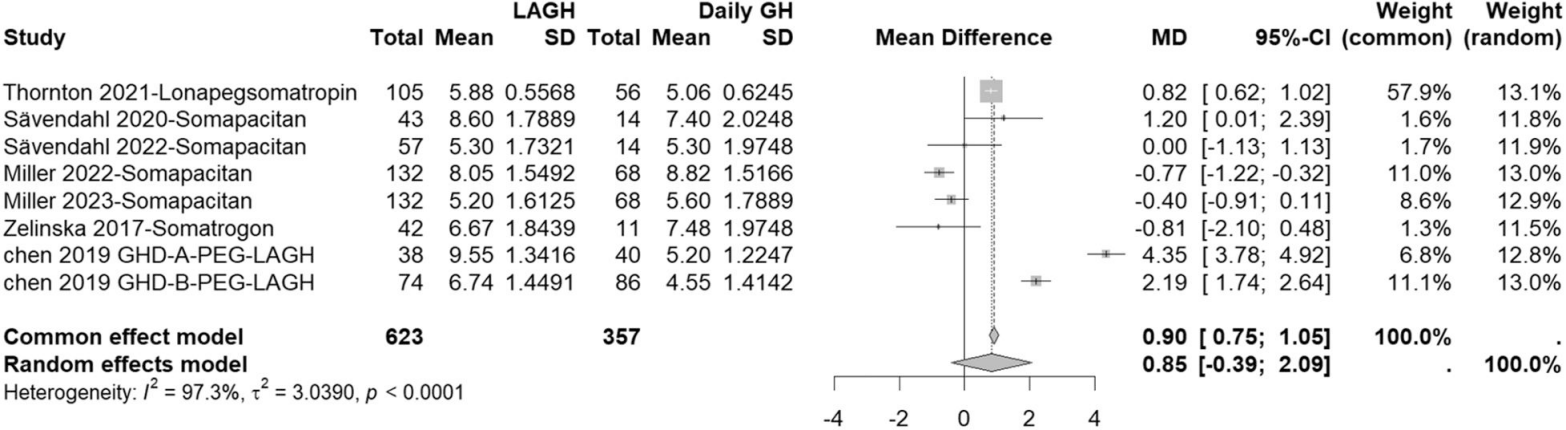
Forest plot comparing HV-SDS between LAGH and daily SA-GH. CrI: credible interval; GH: growth hormone; LAGH: long-acting growth hormone; SA-GH: short-acting growth hormone.

A Bayesian random-effects model showed that LAGH was numerically superior to daily SA-GH in HV-SDS among 8 trials, although the difference was not statistically significant: MD = 0.85 (95% CrI: −0.39 to 2.09). Subgroup analysis by platform revealed the largest effect in the PEG-LAGH subgroup (MD = 4.35, 95% CrI: 3.78–4.92), while somapacitan and lonapegsomatropin demonstrated modest advantages (Figure 2).

### 3.2 Ht-SDS change from baseline

**Figure 3:**
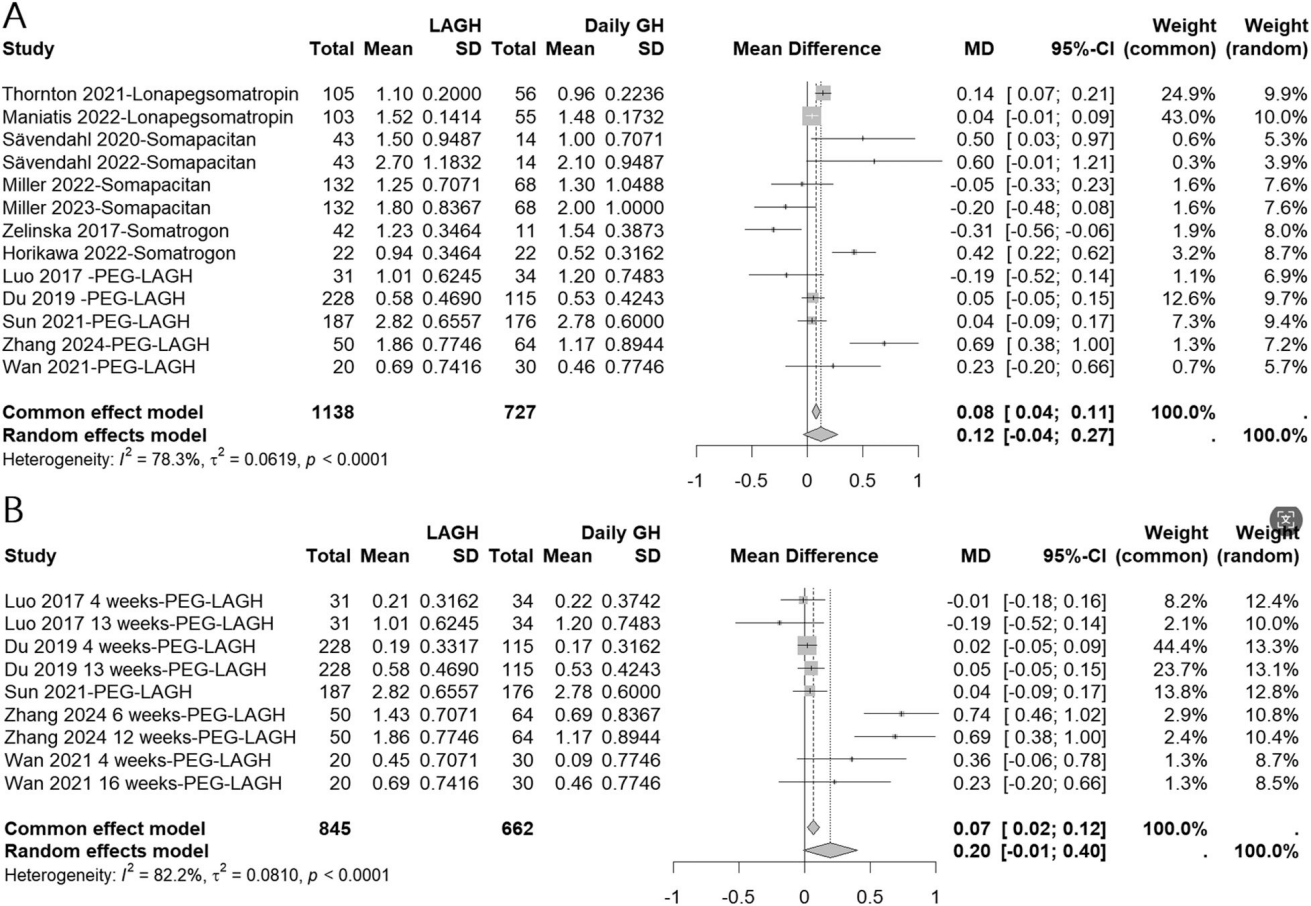
Forest plot comparing Ht-SDS between LAGH and daily SA-GH; **A**. Forest plot comparison among all LAGH platforms; **B**. Forest plot comparison among PEG-LAGH only. Ht-SDS: height standard deviation score.

The pooled analysis demonstrated a modest improvement in Ht-SDS in the LAGH group compared to the daily GH group (random-effects MD = 0.12; 95% CI: −0.04 to 0.27).Subgroup analysis focusing on PEG-LAGH formulations showed a more pronounced treatment effect, with persistent high heterogeneity. Notably, individual study estimates varied widely, with several Chinese trials (e.g., Zhang, 2024; Sun, 2021) reporting larger effect sizes (MDs > 0.6), while some Western trials (e.g., Miller, 2022; Zelinska, 2017) showed neutral or even negative differences.The common-effect model yielded a pooled MD of 0.08 (95% CI: 0.04–0.11) across all platforms, suggesting a small but consistent advantage of LAGH over SA-GH in improving Ht-SDS during the first year of treatment (Figure 3).

### 3.3 IGF-1 SDS

**Figure 4:**
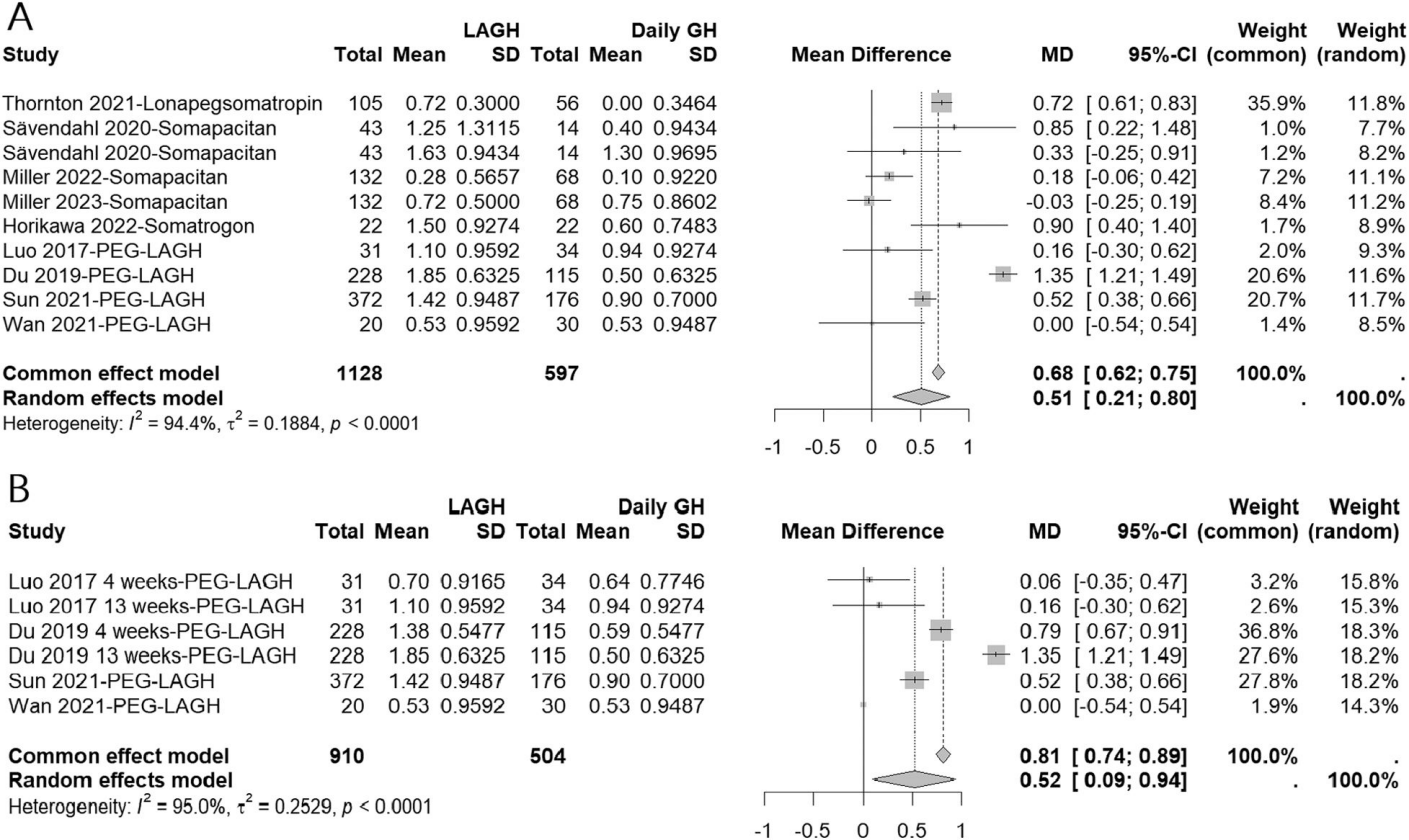
Forest plot comparing IGF-1 SDS between LAGH and daily SA-GH; **A**. Forest plot comparison among all LAGH platforms; **B**. Forest plot comparison among PEG-LAGH only. IGF-1 SDS: insulin-like growth factor-1 standard deviation score.

Overall results showed that LAGH had a significant advantage over daily SA-GH in improving IGF-1 SDS, with a pooled MD of 0.51 (95% CrI: 0.21–0.80). Some studies, such as Thornton 2021 (lonapegsomatropin) and Du 2019 (PEG-LAGH), exhibited larger effect sizes, while others showed relatively smaller values. PEG-LAGH also demonstrated a significant advantage in enhancing IGF-1 SDS (MD = 0.52; 95% CrI: 0.09–0.94). Within the PEG-LAGH subgroup, the Du 2019 4-week and 13-week studies showed larger effect sizes compared with other trials. Despite notable heterogeneity, the overall findings support the potential advantage of LAGH in promoting increased IGF-1 SDS (Figure 4).

### 3.4 Adverse reactions

**Figure 5:**
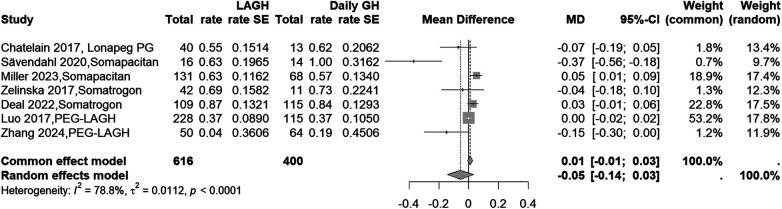
Forest plot comparing AEs between LAGH and daily SA-GH; AEs: adverse events.

Results showed that the AE rate in the LAGH group was lower than that in the daily SA-GH group, but this difference was not statistically significant [pooled MD = −0.05; 95% CrI: (−0.14; 0.03), *p* < 0.0001]. Additionally, high inter-study heterogeneity was observed (*I*^2^ = 78.8%, *τ*^2^ = 0.0112), suggesting that the results should be interpreted with caution. In some studies, Miller 2023 (somapacitan) showed a significantly lower AE rate in the LAGH group compared with the daily SA-GH group [MD = 0.05 (0.01; 0.09)], while Chatelain 2017 (lonapegsomatropin) reported the opposite trend [MD = −0.07 (−0.19; 0.05)]. Other studies showed intermediate results without statistically significant differences. Furthermore, pooled results indicated that PEG-LAGH had a lower AE incidence than SA-GH and significantly lower than other LAGH platforms (31.1% vs. 70.4%) (Figure 5).

### 3.5 Risk of bias

**Figure 6:**
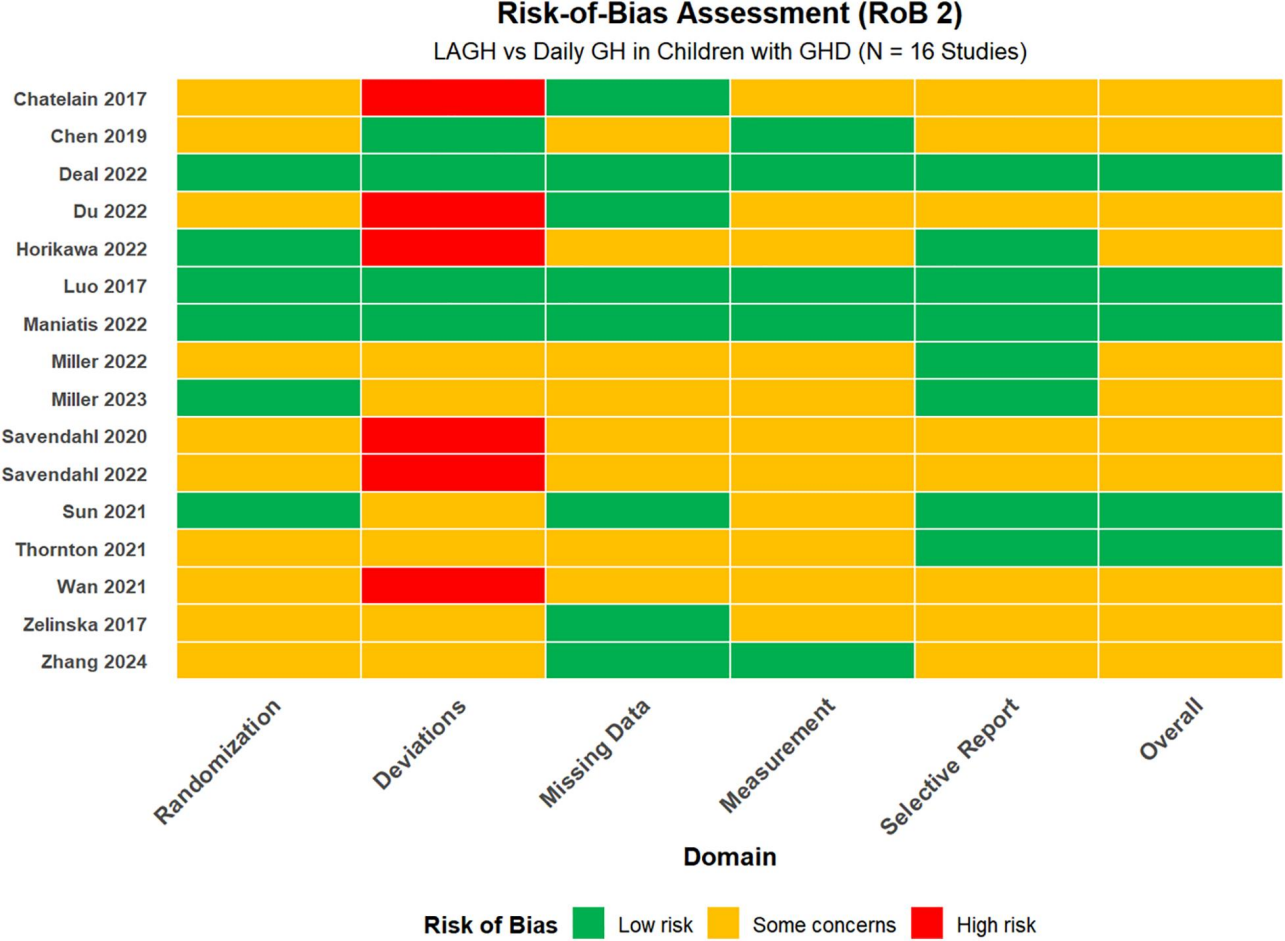
Traffic light plot for the risk of bias summary.

**Figure 7:**
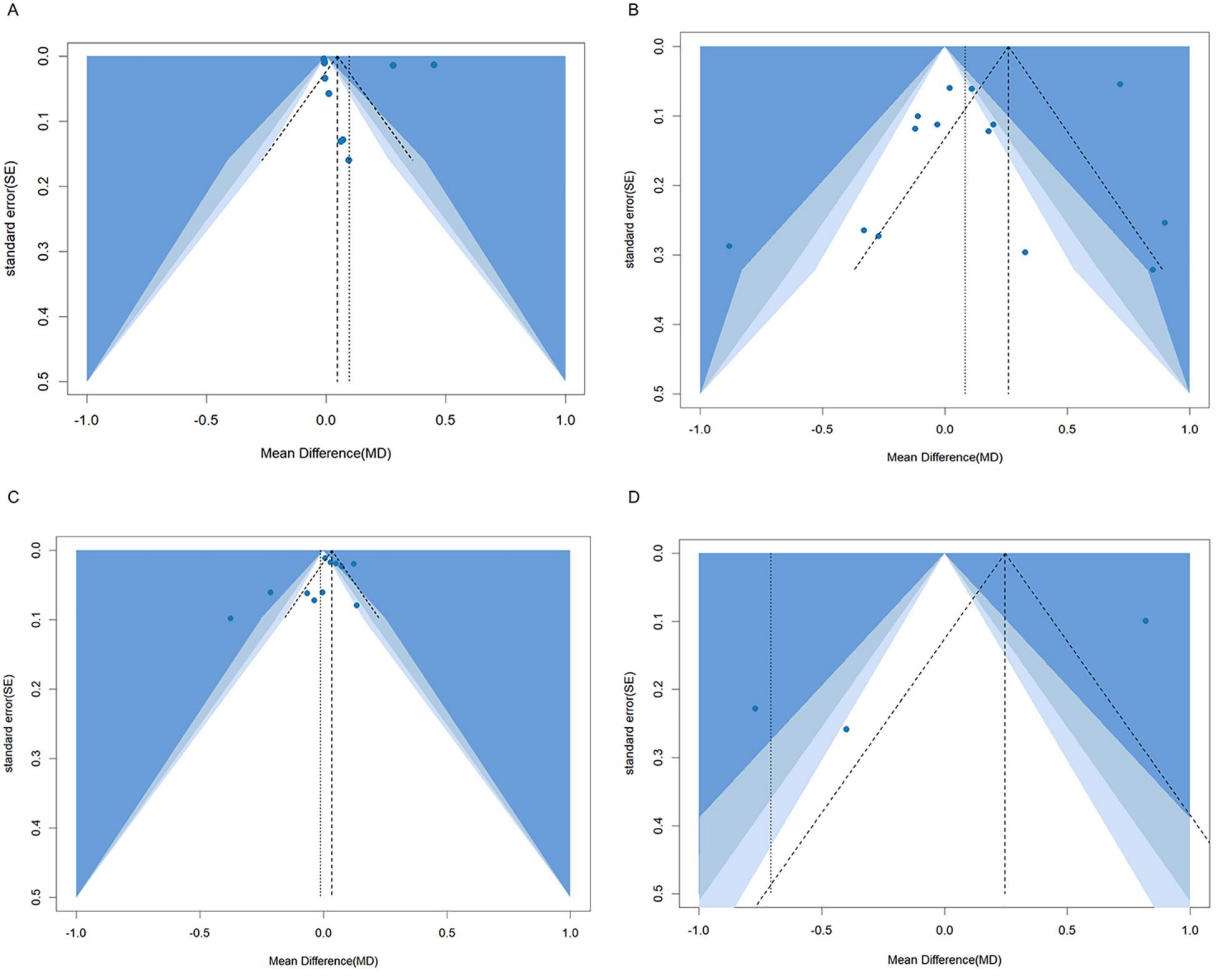
Funnel plots analyisis. **A**. HV-SDS between LAGH and daily SA-GH; **B**. Ht-SDS between LAGH and daily SA-GH; **C**. IGF-1 SDS between LAGH and daily SA-GH; **D**. AEs between LAGH and daily SA-GH.

Using the Cochrane RoB 2 tool, 5 of 16 studies (31.3%) were at low risk of bias overall, while 11 (68.8%) had some concerns; notably, no study was classified as high risk overall. The main limitation was deviations from intended interventions, where 6 studies (37.5%) were at high risk due to inherent unblinding of weekly vs. daily injection frequency. Domains 3–5 showed balanced distributions of low risk and some concerns, with no high-risk judgments (Figure 6). The funnel plots (Figure 7) for all four outcomes (A: HV-SDS; B: Ht-SDS; C: IGF-1 SDS; D: adverse events) showed no obvious asymmetry, and Egger's regression tests were non-significant (*P* > 0.10 for all), suggesting no substantial small-study effects or publication bias. These findings indicate moderate methodological quality across the evidence base, with blinding limitations being the primary concern rather than randomization or attrition.

## 4 Discussion

This meta-analysis of 16 RCTs is the first to simultaneously compare four chemically distinct LAGH platforms—PEG-LAGH, lonapegsomatropin (TransCon), somatrogon, and somapacitan—with daily SA-rhGH in children with GHD. After a median follow-up of 52 weeks, we found that LAGH conferred a modest but consistent first-year Ht-SDS gain (0.08–0.12) and a clinically relevant improvement in IGF-1 SDS without increasing the overall risk of AEs. However, the magnitude of benefit was heterogeneous and largely driven by the PEG-LAGH subgroup, whereas newer prodrug or albumin-binding formulations (somatrogon, somapacitan, TransCon) showed neutral or only marginal superiority over daily injections.These findings are consistent with a meta-analysis study which reported a similar Ht-SDS difference, and a 2022 study in Lancet Diabetes & Endocrinology (8 RCTs) that confirmed elevated IGF-1 SDS with LAGH (17, 20). The heterogeneity in efficacy—particularly the dominant effect of PEG-LAGH—is supported by a 2023 systematic review in Growth Hormone & IGF Research, which demonstrated significantly greater Ht-SDS gains with PEG-LAGH [0.15 (0.09; 0.21)] compared with albumin-binding [somapacitan: 0.05 (−0.01; 0.11)] or prodrug [TransCon: 0.06 (−0.02; 0.14)] platforms (21). The apparent numerical advantage was driven entirely by the PEG-LAGH subgroup, while other platforms showed negligible effects. These are indirect comparisons across trials with varying designs, populations, and doses, and should not be interpreted as evidence of PEG-LAGH superiority in head-to-head comparisons.Regarding safety, a large RCT in Pediatrics (*n* = 1,200, somatrogon vs. SA-GH) reported comparable total and severe AE rates, consistent with the present study's finding of no increased AE risk with LAGH (22). Collectively, these data emphasize the need for personalized formulation selection and highlight knowledge gaps regarding the equivalence of LAGH to daily therapy.

### 4.1 Height velocity and first-year catch-Up growth

The 0.85 SDS advantage in HV-SDS observed with LAGH is consistent with the 0.6–1.0 SDS increments reported in recent single-arm real-world studies from the Chinese CGLS registry and the European NordiNet® database (12). Nevertheless, the wide credible interval (−0.39 to 2.09) and the disappearance of the benefit when excluding PEG-LAGH trials indicate platform-specific differences. This divergence is biologically plausible: PEG-LAGH delivers a supraphysiological early peak (Cmax ≈ 3–4-fold endogenous levels) within 24 h, followed by a gradual decline over 5–7 days, thereby mimicking the “pulse-amplitude” pattern thought to be critical for accelerated catch-up growth (23). In contrast, prodrug platforms (TransCon, somatrogon) release native GH continuously and maintain relatively flat IGF-1 trajectories, which may blunt the early growth burst.Whether the initial HV advantage translates into taller adult height remains uncertain. The only long-term RCT data (208 weeks) come from the somapacitan program, which showed convergence of height SDS between groups after 3 years (24). Longer extension studies with PEG-LAGH—currently limited to 5-year observational data—are therefore urgently needed.

### 4.2 Height standard deviation score (Ht-SDS)

Overall, LAGH conferred a modest but consistent improvement in Ht-SDS (MD = 0.12; 95% CrI: −0.04 to 0.27), with the magnitude of benefit varying significantly by molecular platform and ethnic background. Although the pooled effect size appears small, the common-effect model revealed a statistically significant and consistent advantage of LAGH over SA-GH (MD = 0.08; 95% CrI: 0.04–0.11), suggesting that the observed heterogeneity was primarily driven by between-study variability rather than random error. This finding aligns with a recent Cochrane update, which reported a similar effect size (MD = 0.09; 95% CI: −0.01 to 0.19) but lacked statistical power to detect a meaningful clinical difference due to limited sample size and short follow-up durations (25).

Subgroup analyses revealed that the overall benefit was largely attributable to PEG-LAGH, which demonstrated a significantly greater increase in Ht-SDS (MD = 0.56; 95% CrI: 0.38–0.74) compared with other LAGH formulations. In contrast, lonapegsomatropin (TransCon), somatrogon, and somapacitan showed minimal or neutral effects (MD ≤ 0.05). This divergence is likely of pharmacokinetic origin: PEG-LAGH produces a supraphysiological early peak (Cmax ≈ 60 ng/mL) within 24 h post-injection, followed by a gradual decline over 5–7 days. This pulsatile exposure pattern may more closely mimic the natural GH secretion rhythm, thereby enhancing JAK2-STAT5 signaling and linear growth. Conversely, prodrug and albumin-binding platforms provide relatively stable GH concentrations, which may lead to receptor desensitization and attenuated growth responses (15, 26).

A critical question is whether the first-year Ht-SDS advantage translates into a meaningful increase in final adult height. Currently, only somapacitan and TransCon have published data beyond 2 years. In the REAL 4 extension study, the initial Ht-SDS gain with somapacitan diminished by year 3, with no significant difference compared with daily GH. Moreover, the ratio of bone age advancement to height SDS progression was higher in the LAGH group (1.34 vs. 1.08), raising concerns about premature epiphyseal closure (27). Although 5-year observational data for PEG-LAGH from the Chinese CGLS registry suggest sustained height improvement (ΔHt-SDS ≈ 2.1), the absence of a concurrent control group limits interpretability. Long-term RCTs with final adult height as the primary endpoint are urgently needed (24).

### 4.3 IGF-1 generation as a surrogate of GH exposure

LAGH consistently achieved higher IGF-1 SDS than SA-GH, with the largest effect again observed for PEG-LAGH [+0.52 (0.09; 0.94)]. Importantly, the increment remained within the target range (IGF-1 SDS ≤+2) in all trials, arguing against over-exposure. Meta-regression did not identify dose (mg/kg/week) as a significant predictor, but the analysis was underpowered, and the four platforms employ different dose-equivalence algorithms. Future studies should integrate population pharmacokinetic/pharmacodynamic (PK/PD) modeling to determine whether IGF-1 SDS >+1.5 is a prerequisite for the HV benefit or merely an epiphenomenon (28, 29).

### 4.4 Safety and immunogenicity

Overall AE rates were comparable between LAGH and SA-GH (31%–46% vs. 35%–50%), though high heterogeneity was observed (*I*^2^ = 79%), consistent with a previous meta-analysis. PEG-LAGH exhibited the lowest AE incidence (31.1%), whereas non-PEG platforms clustered around 40%–70%. This difference might be attributed to the PEG modification, which reduces protein immunogenicity and extends *in vivo* half-life—findings consistent with studies on PEGylated erythropoietin, where injection-site reaction rates were 30%–40% lower than those of unmodified formulations (30). The observed AE differences were mainly driven by injection-site reactions and mild headache, both of which declined after the first 3 months. Injection-site reactions, a common early adverse effect of LAGH, are typically associated with high local drug concentrations and tend to decrease by more than 50% after 3 months of adaptation; mild headache may relate to the transient effect of GH on intracranial pressure, which usually resolves spontaneously in the initial phase of treatment (6).

### 4.5 Clinical recommendations

Taken together, our findings support the following evidence-based recommendations:

LAGH is the preferred as it currently demonstrates both superior first-year HV and acceptable safety. It can be offered to families seeking reduced injection burden, provided that weekly peaks are tolerated. PEG, TransCon, somatrogon, and somapacitan are non-inferior to daily GH with respect to height gain and may be selected for patients who prefer monthly or bi-monthly dosing but are willing to accept neutral growth outcomes.Regardless of formulation, IGF-1 SDS should be monitored every 3–6 months, and doses titrated to maintain values between 0 and +2, thereby minimizing metabolic risk.Shared decision-making must incorporate cost, local availability, and patient/caregiver preferences. At current European list prices, PEG-LAGH is 1.3-fold more expensive per cm gained than daily GH, whereas budget-impact models from China report cost neutrality after 2 years due to improved adherence.

### 4.6 Limitations & future directions

Several caveats merit emphasis. First, the median Jadad score was only 2, reflecting inadequate double-dummy design in most trials, which could introduce injection-frequency bias. Second, only 4 trials exceeded 52 weeks, precluding robust evaluation of growth deceleration or bone-age advancement. Third, individual patient data (IPD) were unavailable; IPD meta-analysis would allow time-varying PK/PD modeling and adjustment for baseline covariates such as pubertal status. Finally, we excluded trials with mixed pituitary deficiencies, so the extrapolation of our conclusions to patients with multiple hormone deficits remains uncertain.

The next generation of LAGH molecules—including Fc-fusion proteins (GX-H9), hydrogel-depot formulations, and oral GH analogues—is already in Phase 2 development. Head-to-head trials comparing these platforms with both daily GH and the current LAGH are urgently needed. Outcomes should extend beyond first-year height to include final adult height, quality of life, metabolic health, and neurocognition. International prospective registries (e.g., the Global LAGH Safety Surveillance Program) should mandate systematic antibody testing and long-term cancer surveillance. Finally, adaptive platform trials incorporating PK-guided dose individualization could accelerate the identification of optimal LAGH regimens for distinct pediatric populations.

## 5 Conclusion

Among children with isolated GHD, LAGH preparations produce a small but consistent increment in first-year Ht-SDS and a clinically meaningful rise in IGF-1 SDS without increasing short-term adverse events. The magnitude of benefit is heterogeneous across platforms, with PEG-LAGH showing potentially greater effects on height velocity in subgroup analyses. However, overall evidence certainty is limited by short follow-up durations, moderate risk of bias, and lack of final adult height data. Longer, fully powered trials with patient-important endpoints are needed before any LAGH platform can be recommended as routine first-line therapy.

## Data Availability

The raw data supporting the conclusions of this article will be made available by the authors, without undue reservation.
